# A Series of Eight Cases of Pigment Nephropathy: An Obscured Aspect of Acute Kidney Injury

**DOI:** 10.7759/cureus.64214

**Published:** 2024-07-10

**Authors:** Prem S Patel, Prit P Singh, Archana Archana, Om Kumar

**Affiliations:** 1 Department of Nephrology, Indira Gandhi Institute of Medical Sciences, Patna, IND; 2 Department of Microbiology, Netaji Subhas Medical College and Hospital, Patna, IND

**Keywords:** paroxysmal nocturnal hemoglobinuria, pigment nephropathy, myoglobin cast nephropathy, rhabdomyolysis, hemoglobin cast nephropathy, hemolysis, acute kidney injury

## Abstract

Pigment-induced acute kidney injury (AKI) is an important and preventable complication of rhabdomyolysis or hemolysis. It is characterized by the release of free heme pigment (myoglobin or hemoglobin) in the circulation, leading to direct injury of the proximal tubule and distal tubule obstruction by pigment cast. We are reporting eight cases of pigment-induced AKI, including six cases of myoglobin cast nephropathy and two cases of hemoglobin cast nephropathy. The causes of rhabdomyolysis were strenuous exercise, infection/febrile illness, and drug-induced neuroleptic malignant syndrome. Paroxysmal nocturnal hemoglobinuria and anti-tuberculosis treatment (rifampicin and isoniazid) had led to hemoglobin cast nephropathy each in one case. Seven cases had severe renal failure requiring dialysis. Short-term renal outcome was favorable. However, long-term follow-up is necessary to determine whether pigment-induced AKI has delayed sequelae. Therefore, clinicians should consider rhabdomyolysis or hemolysis as potential hidden causes of AKI in diverse clinical conditions, especially those of non-traumatic origin, to achieve an accurate diagnosis.

## Introduction

Pigment-induced acute kidney injury (AKI) is common and contributes to 7-10% of all cases of AKI [1]. The incidence of pigment-induced AKI in patients with rhabdomyolysis ranges from 10% to 50% [2]. Pigment-induced kidney injury commonly arises from the deposition of either myoglobin or hemoglobin, which are the end results of rhabdomyolysis and hemolysis, respectively. Rhabdomyolysis or hemolysis leads to a sudden release of a high amount of potentially toxic heme pigment (myoglobin or hemoglobin) into the systemic circulation, which after glomerular filtration causes renal injury and renal failure [2,3]. Rhabdomyolysis can occur due to trauma, exertion, or non-exertional causes. Intravascular hemolysis is another important cause of pigment nephropathy. Hemoglobinopathies, paroxysmal nocturnal hemoglobinuria, malaria, transfusion reactions, prosthetic heart valves, and certain drugs are leading causes of intravascular hemolysis [3]. The pathophysiology of pigment-induced AKI is multifaceted, involving vasoconstriction, direct injury to proximal tubular epithelial cells, and obstruction of the distal tubules due to pigment cast formation. Vasoconstriction reduces blood flow to the outer medulla, leading to ischemia. Heme pigment precipitates with the Tamm-Horsfall protein, a process facilitated by acidic urine, forming casts in the distal tubules [2]. This condition is biochemically characterized by elevation of serum creatine phosphokinase, myoglobin levels, lactate dehydrogenase, unconjugated hyperbilirubinemia, and an elevated reticulocyte count. Clinical manifestations of rhabdomyolysis range from an asymptomatic rise in creatine kinase levels to life-threatening AKI. However, patients commonly present with weakness, myalgia, and tea-colored urine. There is no specific treatment for established pigment-induced AKI. The goal of treatment is the prevention of pigment-induced AKI in high-risk patients. Hydration with isotonic saline is the only established preventive measure for pigment-induced AKI. The etiology of pigment-induced nephropathy differs worldwide [2,3]. In India too, the etiology of pigment nephropathy is different in the southern and northern parts [4]. The overall short-term prognosis for pigment-induced AKI is positive, with the majority recovering enough renal function to no longer require dialysis. However, the study has demonstrated an increased risk of chronic kidney disease (CKD) [5]. Thus, the present case series intended to describe the etiology, clinicopathological profile, and outcome of nontraumatic pigment-induced AKI.

## Materials and methods

Study design

This is a retrospective descriptive study. This case series comprises eight cases of biopsy-proven pigment-induced AKI diagnosed between January 2023 and April 2024.

Inclusion and exclusion criteria

The cases of AKI that underwent kidney biopsy between January 2023 and April 2024 were reviewed for evidence of pigment-induced kidney injury. Total eight cases of pigment-induced kidney injury (either myoglobin or hemoglobin-induced nephropathy) with evidence of rhabdomyolysis (elevated serum creatine phosphokinase, lactate dehydrogenase, and myoglobin level) or hemolysis (elevated serum lactate dehydrogenase, elevated unconjugated bilirubin, and elevated reticulocyte count) were included in the analysis. Six cases had myoglobin cast nephropathy and two cases had hemoglobin cast nephropathy. The kidney biopsies in these cases were performed either to confirm or establish the diagnosis in cases of clinically unexplained renal failure.

Data collection

Case files of all patients with myoglobin or hemoglobin cast nephropathy were reviewed. Demographic features, case history, investigation reports, treatment, and outcomes details were recorded. All these details were compiled and presented as case series in the results.

## Results

We analyzed a total of eight cases of pigment-induced AKI diagnosed in the past year. Among these cases, six were associated with rhabdomyolysis, and two were linked to hemolysis caused by paroxysmal nocturnal hemoglobinuria (PNH) and anti-tuberculosis therapy (ATT). Of the six rhabdomyolysis cases, five exhibited elevated serum levels of creatine phosphokinase, lactate dehydrogenase, and myoglobin, while levels were within normal limits in one case. The two hemolysis cases showed elevated serum lactate dehydrogenase, unconjugated hyperbilirubinemia, and an increased reticulocyte count. Among the eight cases, six were young males with a mean age of 27.16 years (range: 17-48). Five had hypertension (mean systolic blood pressure of 155 mmHg and diastolic blood pressure of 95 mmHg), and five experienced oligoanuria. Further details are summarized in Table 1, with individual case descriptions provided below.

**Table 1:** Clinical characteristics, etiology, and outcomes of pigment nephropathy. AKI: acute kidney injury; ATT: anti-tuberculosis therapy; CPK: creatine phosphokinase; LDH: lactate dehydrogenase; HD: hemodialysis; IU/l: international unit per liter; mg/dL: milligram per deciliter; ml/day: milliliter per day; ng/ml: nanogram per milliliter; NMS: neuroleptic malignant syndrome.

S. No.	Age (years)/sex	Blood pressure (mmHg)	24-hour urine output	Clinical syndrome	Serum creatinine (mg/dL)	CPK (IU/L)	LDH (IU/L)	Serum myoglobin (ng/mL)	Precipitating factors	Histological diagnosis	Number of HD sessions	Serum creatinine (mg/dL)	
												Three month	Six months
Case 1	48/male	160/90	<400 ml/day	AKI	6.30	9789	800	560	Anti-psychotic induced NMS	Myoglobin cast nephropathy	7	0.90	0.68
Case 2	26/male	140/100	>400 ml/day	AKI	16.20	1552	418	533.90	Exercise	Myoglobin cast nephropathy	8	0.84	0.60
Case 3	17/male	130/70	<100 ml/day	AKI	22.30	2844	690	1342	Acute febrile illness	Myoglobin cast nephropathy	12	1.00	1.10
Case 4	19/male	136/70	<100 ml/day	AKI	4.46	5467	1650	2406	Exercise	Myoglobin cast nephropathy	6	1.20	0.90
Case 5	18/male	170/100	1000-2000 ml/day	Episodic hematuria	1.72	45	1091	30	Acute febrile illness.	Hemoglobin pigment nephropathy	0	1.00	0.60
Case 6	35/male	150/90	<100 ml/day	AKI	10.06	54466	2020	9908	Acute febrile illness.	Myoglobin cast nephropathy	14	1.12	0.67
Case 7	52/female	134/80	1200-1500 ml/day	AKI	11.49	33	1136	18	ATT (rifampicin and isoniazid)	Hemoglobin pigment nephropathy	7	1.1	1.0
Case 8	34/male	140/80	<100 ml/day	AKI	11.23	84	946	32	Exercise	Myoglobin cast nephropathy	10	Under follow up	

Case 1

**Figure 1:**
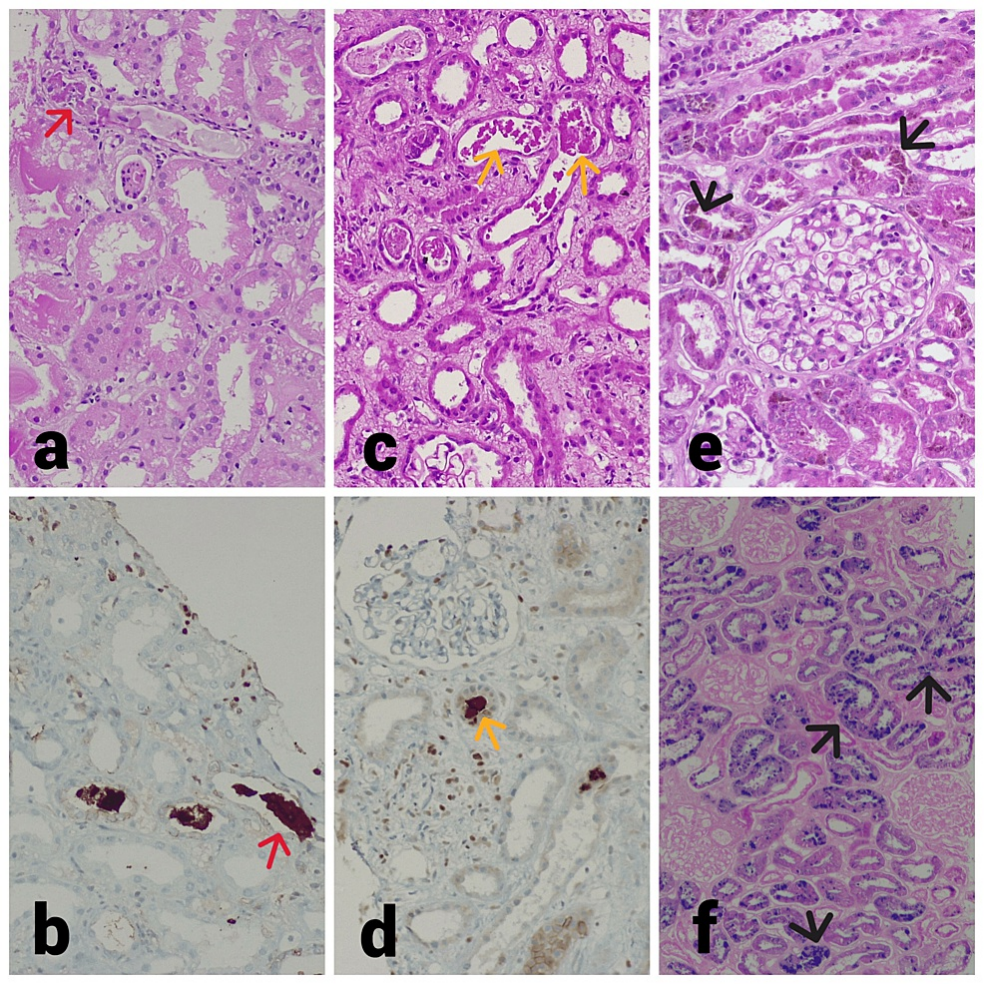
Kidney histology of Case 1, Case 3, and Case 5. (a) Light microscopy shows prominent cytoplasmic vacuolar changes in the tubule, diffuse severe acute injury with epithelial simplification, and loss of brush border. Several variable-sized coarse granular pigmented casts (red arrow) accompanied by inflammatory cell reaction (Case 1, 40x, periodic acid–Schiff). (b) Tubule cast shows myoglobin positive (red arrow) on immunohistochemistry staining (Case 1, 40x, immunohistochemistry). (c) Light microscopy shows prominent cytoplasmic vacuolar changes in the tubule, diffuse severe acute injury with epithelial simplification, and loss of brush border. Scattered variable pigmented granular cast (yellow arrow) is present (Case 3, 40x, hematoxylin and eosin). (d) Tubule cast shows myoglobin positive (yellow arrow) on immunohistochemistry staining (Case 3, 40x, immunohistochemistry). (e) Light microscopy shows non-proliferative glomeruli, diffuse severe acute injury with epithelial simplification and loss of brush border, and several tubules show granular golden-brown (black arrow) pigment deposits (Case 5, 40x, periodic acid–Schiff). (f) Tubule shows intense blue staining (black arrow) on Perls' technique, indicating hemosiderin deposition (Case 5, 40x, Perls' Prussian blue).

A 48-year-old male, diagnosed with schizophrenia and on a regimen of quetiapine 50 mg twice daily and chlorpromazine 100 mg once daily for two years, presented with fever, abnormal body posture, transient loss of consciousness, progressive oliguria, and anasarca over the past five days. He did not have hematuria, lower urinary tract symptoms, or intake of nephrotoxic drugs. A general examination revealed pallor and anasarca. Neurological examination was unremarkable, except for disorientation and rigidity of extremities and trunk. Investigation showed renal failure and rhabdomyolysis (Table 1). A urine dipstick test was positive for blood. A kidney biopsy was performed to confirm the diagnosis of pigment-induced AKI. Renal histology revealed non-proliferative glomeruli, diffuse severe acute tubular injury with coarse granular, and pigmented cast. The cast was positive for myoglobin and negative for hemoglobin on immunohistochemistry (Figure 1). Direct immunofluorescence was negative. Hence, the diagnosis of myoglobin cast nephropathy was made. Neuroleptic drugs were discontinued, and oral bromocriptine 2.5 mg twice a day and trihexyphenidyl 2 mg daily were given for two weeks, along with intravenous fluid, hemodialysis, and symptomatic treatment. He received seven hemodialysis sessions, his urine output progressively increased, and serum creatinine normalized by the second week.

Case 2

A 26-year-old male presented with pain in both thighs, followed by bilateral flank pain and swelling over both feet for three days, after strenuous exercise. He did not have a history of hematuria, oliguria, lower urinary tract symptoms, or intake of nephrotoxic drugs. A general examination was unremarkable except for pedal edema. The systemic examination was normal. A urine analysis was normal. The hemogram was normal. Investigation showed renal failure and rhabdomyolysis. Clinically, his renal failure was unexplained. Thus, a kidney biopsy was performed (Table 1). Renal histology revealed non-proliferative glomeruli and diffuse severe acute tubular injury with coarse, granular, and myoglobin-positive pigmented cast. Direct immunofluorescence was negative. Thus, a diagnosis of myoglobin cast nephropathy was made. He was managed with intravenous fluid, eight hemodialysis sessions, and symptomatic treatment. Urine output progressively increased and serum creatinine normalized by the third week.

Case 3

A 17-year-old male factory worker presented with a fever associated with chills for 10 days, followed by abdominal pain and decreased urine output for five days. The fever was continuous and not associated with cough, icterus, lower urinary tract symptoms, hematuria, headache, convulsion, and visual disturbance. He did not give a history of nephrotoxic drug use. A general examination revealed pallor and anasarca. The systemic examination was normal. A urine examination was unremarkable. The hemogram showed normocytic normochromic anemia. Investigation showed renal failure and rhabdomyolysis. Workups for malaria, Leptospira, and scrub typhus were negative. Blood and urine cultures did not show any growth. Serum procalcitonin was normal. Clinically, his renal failure was unexplained, and a kidney biopsy was performed to establish an accurate diagnosis (Table 1). Renal histology revealed non-proliferative glomeruli and diffuse severe acute tubular injury with coarse, granular, and pigmented cast. The cast was positive for myoglobin and negative for hemoglobin on immunohistochemistry (Figure 1). Direct immunofluorescence was negative. Thus, the diagnosis of myoglobin cast nephropathy was confirmed. He was managed with empirical intravenous meropenem (500 mg twice a day), intravenous fluid, 12 hemodialysis sessions, and symptomatic treatment. The fever gradually subsided and the patient became afebrile after seven days of antibiotic therapy. His urine output also progressively increased and serum creatinine normalized by the fourth week.

Case 4

A 19-year-old male working in a saree factory under hot and humid conditions presented with nausea and reduced urine output for seven days. He did not give a history of hematuria, lower urinary tract symptoms, or intake of nephrotoxic drugs. A general and systemic examination was unremarkable, except for pedal edema. A urine analysis was normal. The hemogram was normal. Investigation showed renal failure and elevated serum creatine phosphokinase and lactate dehydrogenase (Table 1). A kidney biopsy was performed to confirm the diagnosis of pigment nephropathy. Renal histology revealed non-proliferative glomeruli and diffuse severe acute tubular injury with coarse, granular cast. Direct immunofluorescence was negative. Thus, based on clinical, laboratory, and histological evidence, a diagnosis of myoglobin-induced nephropathy was made. He was managed with intravenous fluid, six hemodialysis sessions, and symptomatic treatment. Urine output progressively increased and serum creatinine normalized by the third week.

Case 5

An 18-year-old male student presented with multiple episodes of self-resolving red color urine for two years. It was not associated with a sore throat, cough, lower urinary tract symptoms, joint pain, or skin rash. He did not give a history of nephrotoxic drugs. Hematuria used to get precipitated by febrile illness or exertion and resolved with rest. A general examination revealed pallor. His blood pressure was 160/90 mm of Hg. The systemic examination was normal. The hemogram showed hemolytic anemia. The biochemical result showed mild renal failure and elevated lactate dehydrogenase (1091 IU/L) (Table 1). A urine dipstick test was positive for blood. Serum antinuclear antibody (ANA) and complement were normal. A provisional clinical diagnosis of IgA nephropathy was made, and a kidney biopsy was performed to confirm the diagnosis. Renal histopathology revealed non-proliferative glomeruli and diffuse severe acute tubular injury with many granular golden-brown pigments cast, which stained intensely blue with Perls' technique. Direct immunofluorescence study was negative for immune deposits (Figure 1). Thus, the kidney biopsy changed the diagnosis to hemoglobin pigment-induced AKI. Hemolysis workup revealed a PNH clone within RBC (72.86% CD59 deficiency), granulocyte (98.62% CD24/CD55 deficiency), and monocyte (99.35% CD14/CD55 deficiency). Renal artery color Doppler revealed left renal artery stenosis. Finally, the diagnosis of PNH-induced hemoglobin pigment-induced AKI, associated with left renal artery stenosis, was confirmed. He was managed with intravenous fluid, oral nifedipine, and symptomatic treatment. Urine color improved and serum creatinine became normal by a week.

Case 6

**Figure 2:**
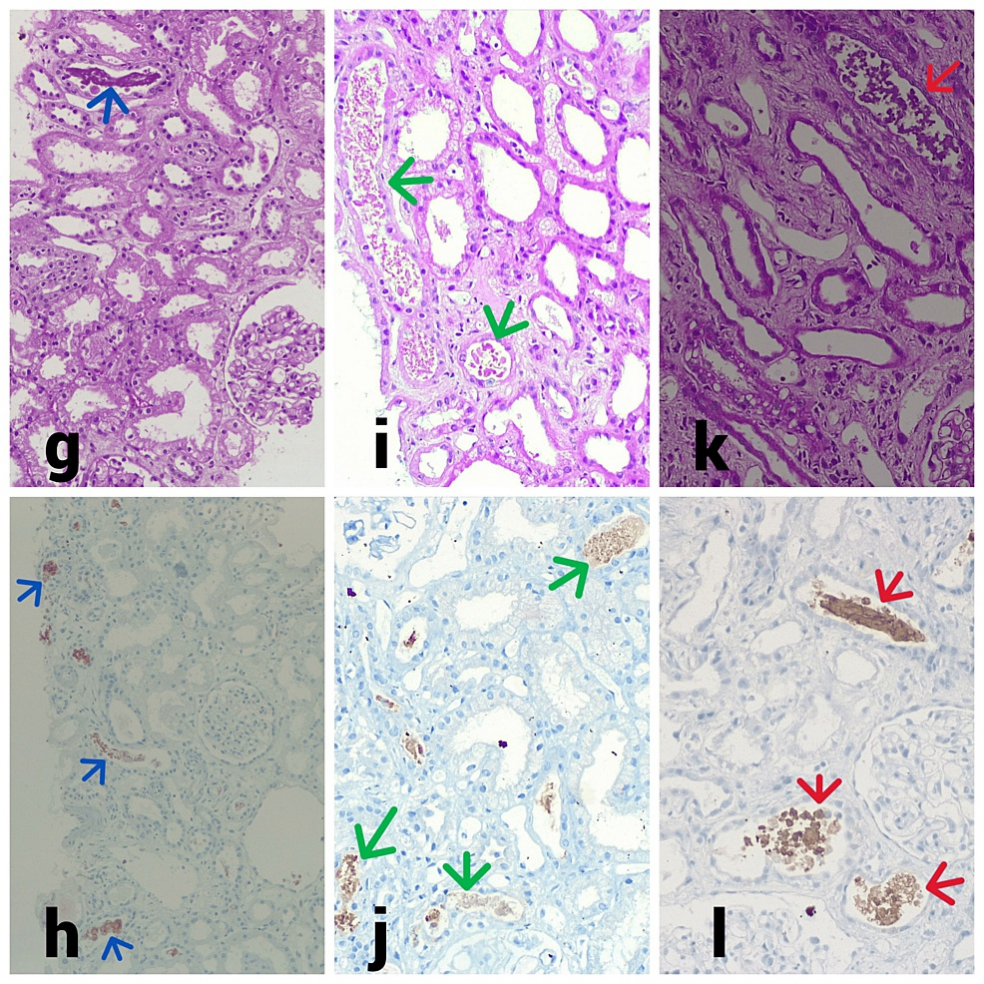
Kidney histology of Case 6, Case 7, and Case 8. (g) Light microscopy shows non-proliferative glomeruli, tubule shows prominent cytoplasmic vacuolar changes, diffuse severe acute injury with epithelial simplification, and loss of brush border. Scattered pigmented granular cast (blue arrow) are present (Case 6, 40x, periodic acid–Schiff). (h) Tubule cast shows myoglobin positive (blue arrow) on immunohistochemistry staining (Case 6, 40x, immunohistochemistry). (i) Light microscopy shows prominent cytoplasmic vacuolar changes in the tubule, diffuse severe acute injury with epithelial simplification, and loss of brush border. Several variable-sized coarse granular pigmented casts (green arrow) (Case 7, 40x, periodic acid–Schiff). (j) Tubular cast shows hemoglobin positive (green arrow) on immunohistochemistry staining (Case 7, 40x, immunohistochemistry). (k) Light microscopy shows prominent cytoplasmic vacuolar changes in the tubule, diffuse severe acute injury with epithelial simplification, and loss of brush border. Scattered pigmented granular cast (red arrow) are present (Case 8, 40x, periodic acid–Schiff). (l) Tubule cast shows myoglobin positive (red arrow) on immunohistochemistry staining (Case 8, 40x, immunohistochemistry).

A 35-year-old male farmer presented with a self-resolving low-grade fever for five days, followed by decreased urine output and generalized swelling for five days. The fever was continuous and not associated with cough, lower urinary tract symptoms, hematuria, joint pain, and skin rash. He did not have a history of nephrotoxic drugs. A general examination revealed pallor and anasarca. The systemic examination was normal. The 24-hour urine output was less than 100 ml. A urine examination was inconclusive. The hemogram showed normocytic normochromic anemia. The biochemical result showed renal failure and elevated serum creatine phosphokinase and lactate dehydrogenase. Serum procalcitonin, erythrocyte sedimentation rate (ESR), and CRP were normal. The immunological workup was negative. Blood and urine cultures did not show any growth (Table 1). Clinically, the cause of renal failure was not clear, and a kidney biopsy was performed. Renal histology revealed non-proliferative glomeruli and diffuse severe acute tubular injury with several, granular, myoglobin-positive pigment casts (Figure 2). Direct immunofluorescence was negative. Hence, the diagnosis of myoglobin cast nephropathy was made. He was managed with intravenous fluid, 14 hemodialysis sessions, and symptomatic treatment. His urine output progressively increased and serum creatinine normalized by the fourth week.

Case 7

A 52-year-old female with diabetes and hypertension for four months presented with abdominal pain and renal dysfunction of 10 days duration. She had a low-grade undocumented fever and was taking ATT (rifampicin, isoniazid, pyrazinamide, and ethambutol) for a week, as advised empirically by a local doctor. She did not have a fever, lower urinary tract symptoms, hematuria, joint pain, or skin rash on admission. She did not have a history of nephrotoxic drugs. A general examination was unremarkable, except for pallor. Her blood pressure was 150/100 mmHg. The systemic examination was normal. The 24-hour urine output was 1200-1500 ml. Dip-stick urine examination was positive for blood without RBC on microscopy. The hemogram showed normocytic normochromic anemia with increased reticulocyte counts. The biochemical result showed renal failure and elevated lactate dehydrogenase. Serum creatine phosphokinase, myoglobin, procalcitonin, ESR, and CRP were normal. The immunological and tuberculosis workup was negative. Blood and urine cultures were also negative (Table 1). Provisionally, it appeared that the case was rifampicin-induced acute interstitial nephritis, and a kidney biopsy was performed to confirm the diagnosis. Renal histology revealed non-proliferative glomeruli and diffuse severe acute tubular injury with several, granular, hemoglobin-positive pigment casts (Figure 2). Direct immunofluorescence was negative. Thus, the kidney biopsy helped us reach the correct diagnosis of rifampicin-induced hemoglobin cast nephropathy. ATT was discontinued. She was managed with intravenous fluid, seven hemodialysis sessions, and symptomatic treatment. Her renal function gradually improved, hemodialysis was discontinued and serum creatinine normalized by the fourth week.

Case 8

A 34-year-old male farmer presented with acute onset low-grade fever and pain in the bilateral calf and thigh after working in the field for a day. The fever was continuous and not associated with cough, lower urinary tract symptoms, hematuria, joint pain, and skin rash. He did not have a history of nephrotoxic drugs. He received symptomatic treatment for three to four days from a local doctor without much relief of calf and body pain. Subsequently, he noticed decreased urine output and generalized swelling for eight days. At presentation, the general examination was normal except for anasarca. The systemic examination was unremarkable. The 24-hour urine output was less than 100 ml. A urine examination was inconclusive. The hemogram was normal. The biochemical result showed renal failure and elevated serum creatine phosphokinase and lactate dehydrogenase. Serum procalcitonin, ESR, and CRP were normal. The immunological workup was negative. Blood and urine cultures were sterile (Table 1). A kidney biopsy was performed to establish the cause of renal failure. Renal histology revealed non-proliferative glomeruli and diffuse severe acute tubular injury with several, granular, myoglobin-positive pigment casts (Figure 2). Direct immunofluorescence was negative. Hence, the diagnosis of myoglobin cast nephropathy was made. He was managed with intravenous fluid, 10 hemodialysis sessions, and symptomatic treatment. His urine output progressively increased and serum creatinine normalized by the sixth week. He is currently under follow-up.

## Discussion

AKI is a serious manifestation of the harmful effect of heme pigment on the kidney [4]. Pigment-induced kidney injury accounts for 7-10% of all cases of AKI [1]. Rhabdomyolysis occurs when the energy supply to the muscle is insufficient to meet demands, particularly during prolonged strenuous or unaccustomed activity or exertion under hot and humid conditions where muscle energy production is impaired. The percentage of rhabdomyolysis cases complicated by AKI ranges widely, from 15% to over 50% [2]. This variability in reported AKI incidence likely stems from differences in the severity of rhabdomyolysis and definitions of AKI. Similarly, the incidence of AKI due to hemolysis shows variability. Nowadays, AKI is more often multifactorial and less likely to be solely attributed to hemolysis alone [3]. The mechanism of pigment-induced AKI is multifaceted, involving vasoconstriction, direct injury to proximal tubular epithelial cells, and obstruction of the distal tubules due to pigment cast formation. Vasoconstriction reduces blood flow to the outer medulla, leading to ischemia. Heme pigment precipitates with the Tamm-Horsfall protein, a process facilitated by acidic urine, forming casts in the distal tubules. We analyzed a total of eight cases of pigment-induced AKI diagnosed in the last year. The kidney biopsies in these cases were performed either to confirm the clinical suspicion or to establish the diagnosis in cases of clinically unexplained renal failure. Six cases had rhabdomyolysis and two had hemolysis caused by PNH and ATT. Out of eight cases, six were young males (mean age: 27.16 years, range: 17-48), five had hypertension (mean systolic blood pressure of 155 mmHg and diastolic blood pressure of 95 mmHg), and five had oligoanuria. The patient with PNH had left renal artery stenosis. Risk factors for rhabdomyolysis include age <10 years and >60 years, male, Black race, body mass index >40 kg/m2, low premorbid physical fitness, and dehydration [6-8]. Likewise, our findings that pigment-induced AKI occurred commonly in male patients and rhabdomyolysis caused by strenuous work in the hot and humid environment of the factory also agree with another study [6-8]. Rhabdomyolysis is multifactorial and characterized by the release of muscle cell constituents into the circulation. As a consequence, serum myoglobin, serum creatine phosphokinase (CPK), and lactate dehydrogenase (LDH) rise [7]. Myoglobin is a 17.8-kDa nephrotoxic protein and it causes renal vasoconstriction and proximal tubular cytotoxicity and can precipitate in the distal tubules leading to obstruction [2]. In our study, rhabdomyolysis was caused by drug-induced neuroleptic malignant syndrome (one case), strenuous exercise (two cases), and infection/febrile illness (two cases). Serum CPK (mean of 1235.3 IU/L, ranging from 1552 to 54466), serum LDH (mean of 1111.5 IU/L, ranging from 418 to 2020), and serum myoglobin (mean of 2950 ng/mL, ranging from 533 to 9908) were elevated in five cases with rhabdomyolysis. Though serum myoglobin levels rise earliest after muscle injury, its metabolism is rapid and unpredictable. Hence, the measurement of serum myoglobin has a low sensitivity for the diagnosis of rhabdomyolysis [9]. Thus, serum CPK is the most sensitive enzyme marker of muscle injury. The etiology of rhabdomyolysis is geographically diverse and can be traumatic (crush syndrome or immobilization), nontraumatic exertional (exertion, eccentric exercises, hyperthermia, or metabolic and other myopathies), and nontraumatic non-exertional (drugs or toxins, infections, or electrolyte disorders) [10]. In our study, rhabdomyolysis was nontraumatic exertional (three cases) and nontraumatic non-exertional (infection: two cases; drug: one case) in nature.

Intravascular hemolysis is another important cause of pigment nephropathy. PNH, hemoglobinopathies, malaria, transfusion reactions, prosthetic heart valves, and drugs are leading causes of intravascular hemolysis [3,11-14]. In one case, PNH led to hemolysis precipitated by self-resolving febrile illness. In another case, probably ATT (rifampicin and isoniazid) led to hemolysis and hemoglobin pigment-induced AKI. Another study also reported the case of rifampicin-induced hemoglobin cast nephropathy [14]. Envenomation, poisonings, malaria, infections, and sepsis are the common etiologies for both rhabdomyolysis and hemolysis [3,13]. We did not find envenomation or poisoning as a cause of pigment-induced AKI in our study, which is a more prevalent cause in the southern part of India.

There is no morphological difference in the tubular injury and pigment cast due to rhabdomyolysis or hemolysis [3]. In our study, all cases had severe acute tubular necrosis with granular pigment cast and we did not notice any morphological difference in renal histology (Figure 1). Thus, morphologically, it is not possible to differentiate the cause of pigment-induced AKI. Many times, staining by Perls' technique points toward hemolysis as the cause of AKI [3]. In one case, Perls' staining of tissue helped us diagnose hemoglobin pigment-induced AKI. Hence, in addition to clinical and laboratory evidence, the immunohistochemistry for myoglobin or hemoglobin would help in the diagnosis.

The major management goal is the prevention of pigment-induced AKI in at-risk (CPK >5000 IU/L) patients [6]. In our study, seven out of eight cases presented with established AKI, thus prevention was not possible. There is no specific treatment for established pigment-induced AKI, and only supportive treatments like maintenance of fluid and electrolyte balance and tissue perfusion, and initiation of dialysis when necessary are worthy. Seven out of eight cases had severe renal failure (mean serum creatinine of 10.43 mg/dl) and received hemodialysis (mean of nine sessions of hemodialysis). These findings indicate that pigment-induced AKI occurs commonly in young adults, and often presents with oligoanuria and renal failure requiring hemodialysis.

The short-term prognosis for pigment-induced AKI is generally positive, with most patients recovering enough renal function to discontinue dialysis [5,15]. However, studies have shown an elevated risk of CKD regardless of initial renal recovery. In a study by Sakthirajan et al., five out of 46 patients developed CKD [15]. Another study by Liapis et al. reported CKD in 45% of their cohort, with 18% of those affected succumbing to the illness [16]. In the present study, the renal function sufficiently recovered in all patients within one to four weeks (mean of 3.3 weeks) and became dialysis independent. Serum creatinine was within the normal range at three and six-month follow-up.

Our study had several important limitations. Being a case series with a limited number of cases, the findings cannot be extrapolated to the general population. Additionally, the limited duration of follow-up in the study makes it difficult to draw conclusions about long-term outcomes.

## Conclusions

Pigment-induced AKI has a varied etiology and often leads to renal failure requiring hemodialysis. Therefore, clinicians should consider rhabdomyolysis or hemolysis as potential hidden causes of AKI in diverse clinical conditions, especially those of non-traumatic origin, to achieve an accurate diagnosis. Short-term renal outcomes were favorable. However, long-term follow-up is necessary to determine whether pigment-induced AKI has delayed sequelae.
